# The Spillover of US Immigration Policy on Citizens and Permanent Residents of Mexican Descent: How Internalizing “Illegality” Impacts Public Health in the Borderlands

**DOI:** 10.3389/fpubh.2015.00155

**Published:** 2015-06-11

**Authors:** Samantha Sabo, Alison Elizabeth Lee

**Affiliations:** ^1^Department of Health Promotion Sciences, Zuckerman College of Public Health, The University of Arizona, Tucson, AZ, USA; ^2^Departamento de Antropología, Universidad de las Américas Puebla, Cholula, Mexico

**Keywords:** immigration policy, mistreatment, border health, stress, psychological, prevention and control

## Abstract

**Background:**

The militarization of the US–Mexico border region exacerbates the process of “Othering” Latino immigrants – as “illegal aliens.” The internalization of “illegality” can manifest as a sense of “undeservingness” of legal protection in the population and be detrimental on a biopsychological level.

**Objective:**

We explore the impacts of “illegality” among a population of US citizen and permanent resident farmworkers of Mexican descent. We do so through the lens of immigration enforcement-related stress and the ability to file formal complaints of discrimination and mistreatment perpetrated by local immigration enforcement agents, including local police authorized to enforce immigration law.

**Methods:**

Drawing from cross-sectional data gathered through the National Institute of Occupation Safety and Health, “Challenges to Farmworker Health at the US–Mexico Border” study, a community-based participatory research project conducted at the Arizona–Sonora border, we compared Arizona resident farmworkers (*N* = 349) to Mexico-based farmworkers (*N* = 140) or Transnational farmworkers who cross the US–Mexico border daily or weekly to work in US agriculture.

**Results:**

Both samples of farmworkers experience significant levels of stress in anticipation of encounters with immigration officials. Fear was cited as the greatest factor preventing individuals from reporting immigration abuses. The groups varied slightly in the relative weight attributed to different types of fear.

**Conclusion:**

The militarization of the border has consequences for individuals who are not the target of immigration enforcement. These spillover effects cause harm to farmworkers in multiple ways. Multi-institutional and community-centered systems for reporting immigration-related victimization is required. Applied participatory research with affected communities can mitigate the public health effects of state-sponsored immigration discrimination and violence among US citizen and permanent residents.

## Introduction

US immigration enforcement efforts grew considerably over the last few decades, with a nearly 15-fold increase in funding from 1986 to 2012 channeled into the nation’s principle enforcement agencies. Customs and border protection (CBP) and immigration and customs enforcement (ICE), whose FY2012 budget totaled 17.9 billion dollars (1), contribute to the militarization of the US–Mexico border. Militarization is defined as the saturation of and pervasive encounters with immigration officials including local police enacting immigration and border enforcement policy with military style tactics and weapons (2). These enforcement measures are applied at ports of entry (POE), in the deserts, rivers, and mountains between POEs, and, increasingly, in public spaces, workplaces, and residential areas in the border region and elsewhere (3, 4).

The territorial boundary of the sovereign state has always been fundamental to the creation of social hierarchies. The intersections of ethnicity, race, class, and gender relegate people to social categories some of whose members have rights of membership, including US citizens and permanent residents and “Others” who do not possess such rights, such as unauthorized immigrants or “illegal aliens” (5, 6). In the US–Mexico border region, the process of “Othering” categorizes Latino immigrants and migrants, including their non-immigrant co-ethnics as “illegal aliens” (5, 7, 8). The symbolic violence (9, 10) or the implicit way in which cultural and social domination is maintained on an unconscious level through discriminatory practices generated by sexism, racism, and classism naturalizes the notion of “illegality.” Through this process, certain groups are categorized as non-rights-bearing individuals (11, 12). The erasure of legal personhood manifests as the inability to obtain work authorization and restricted physical and social mobility, which reinforces immigrants’ forced invisibility, exclusion, and sense of vulnerability to being deported (12, 13). The militarization of the border contributes to the construction of such notions of “illegality” of Latino populations by inscribing difference “upon Mexican migrants” themselves, as their distinctive spatialized (and racialized) status as “illegal aliens,” as Mexicans “out of place” (5).

In the context of the “War on Terror,” the regulatory policies associated with enforcement conflate migrants with terrorists, drug smugglers, and human traffickers who represent a threat to national security (14–16). The criminalization of immigration law erodes the legal protections that once covered non-citizens, subjecting ever-growing numbers to deportation (17–19). Further, there is growing evidence that border enforcement leads to maltreatment of persons that violates their civil and human rights through the excessive use of force and verbal and physical abuse (4, 14, 20).

Cumulative exposure to institutional arrangements, ethno-racial hierarchies, and citizen/non-citizen distinctions that systematically marginalize individuals create disproportionate levels of structural vulnerability (21). Defined as “a positionality that imposes physical and emotional suffering on specific population groups and individuals in patterned ways,” structural vulnerability reproduces inequality by casting certain groups as less worthy of material and social protection (22). The subordinated status created through “illegality” may be internalized by Latino immigrant and migrants and detrimental on a biopsychological level (23–26). Farmworkers especially experience high levels of structural vulnerability due to their subordinate status in the social hierarchy (27). As a result, farmworkers in general experience greater prevalence of chronic disease risk factors and poorer mental health outcomes compared to non-farmworker US Hispanic populations (28–31).

This study aimed to explore ways in which a relatively large sample of immigrant and migrant farmworkers of Mexican descent who are US citizen and permanent residents and live and work in the Arizona–Sonora, Mexico border region experience “illegality” and the impact it has on their health. We hypothesized that transnational border residents, or those farmworkers who live permanently in Mexico and cross the border to work in US agriculture would be more likely to experience an internalized sense of “illegality” due to their residence outside the US and the need to cross the border for employment. Such perceptions of “illegality” could come in form of feeling as though they “belonged less” to the nation compared to those immigrant and migrant farmworkers who live in Arizona because of their residence outside the country. We contend that the need to cross the border daily and interact frequently with immigration enforcement officials at points of entry would bolster transnational farmworkers sense of being “Other” and negatively affect their well-being.

## Materials and Methods

The National Institute of Occupation Safety and Health, “challenges to farmworker health (CFH) at the US–Mexico Border” is a community-based participatory research (CBPR) project conducted by the University of Arizona, Zuckerman College of Public Health, and the Binational Migration Institute located in the Department of Mexican American Studies in partnership with *Campesinos Sin Fronteras*, a community-based agency serving regional border residents and *Derechos Humanos*, a human rights organization advocating on behalf of Arizona immigrant families (32). A detailed discussion of this partnership is reported elsewhere (4, 33).

Challenges to farmworker health is a cross-sectional, population-based survey using a randomized proportionately representative household sample (*N* = 299) and a convenience sample (*N* = 200) of men and women of Mexican descent aged 20 years and older who were farmworkers during the 12 months preceding the survey. To obtain the household sample, researchers randomly selected census blocks for three adjacent Arizona-border communities; all were low income and typically medically under-served communities in which agricultural workers were the dominant residents. A modified survey was then utilized as an opportunistic survey conducted at specific pick-up points for farmworkers with the same enrollment restrictions mentioned, who may have been missed in the primary survey. This survey targeted those farmworkers not living in local household but rather commute from a distance, live in their automobiles, live across the border (including US residents), or live in “colonias” not yet mapped to the existing city and county neighborhood plots. For the purposes of this paper, the household and opportunistic samples were merged and stratified by transnational farmworkers who did not live in the US but crossed the Mexico border daily or weekly to work in US agriculture (*N* = 140) and those farmworkers whose primary residence was in Arizona (*N* = 349) referred to herein as Arizona-based farmworkers.

Essential to this study, were community health workers or *Promotoras*, who shared cultural and linguistic history of participants, contributed to survey modification and provided insight into cultural and regional relevance of interview questions. *Promotoras* were trained by UA research staff to conduct interviews and collected the majority of the survey data over the summer months of 2006–2007. *Promotoras* contacted a total of 323 adults who met study criteria, of which 299 agreed to participate, resulting in a 93% response rate. We believe CBPR, which equitably engaged affected community members throughout the research process, and the full engagement of *Promotoras* as trusted members of the community, increased the likelihood of participation and quality of the self-reported data. A detailed description of the CFH study sampling frame and partner agency relationships in CBPR is found elsewhere (33).

To examine the existing level of structural vulnerability within the population, descriptive statistics were calculated for variables shared by both the household and the abbreviated opportunistic survey instruments, these include selected demographics (age, years working in US agriculture), immigration status, access to health care coverage, and immigration encounter and immigration-related stress. Drawing from survey items from the Immigration and Border Interaction Survey conducted over a 15-year period in one Southern Arizona-border community (34, 35), respondents were also asked about their experiences with immigration officials and the perceptions of how immigration officials differentiate between US citizens and individuals unauthorized to be in the US. Stress was measured with items from the Border Community and Immigration Stress Scale (BCISS), a 21-item scale that considers the presence and intensity of culturally and contextually relevant stressors (33). BCISS stress domains include migration, acculturation, and barriers to health care, discrimination, economic strains, and family separation. For this study, we explored four border community and immigration-related stressors, including stress caused by encounters with immigration officials, local police, and the presence of military in the region. The BCISS is a 5-point Likert scale, which measures the level or intensity of the stress experienced for each given domain. For the domains of interest, we created a dichotomous variable to categorize respondents by self-reported feelings of very or extreme stress and those that experienced low to moderate stress. Data reported here illustrate those respondents who self-reported very or extreme stress, which is narrated in text as intense stress. Full description of the 21-BCISS can be found elsewhere (33).

Most importantly, we wanted to explore how such cumulative immigration-related surveillance, encounters and stress might contribute to a sense of undeservingness of social protection from immigration-related mistreatment or discrimination among study participants. To do so, we analyzed Arizona and transnational participants’ short narratives of reasons to file and not to file a formal complaint with immigration authorities regarding an immigration related mistreatment episode.

### Analysis

We explored differences between the two samples through Fisher’s Exact for demographic and experiences with immigration officials. All statistical analyses were performed using STATA 10.0 software. We used grounded theory to code themes that emerged from the short narratives and stratified that analysis by Arizona and transnational participants (36). The UA Office of Human Subject Protection approved this research.

## Results

There were no significant differences between the two samples in terms of immigration status as approximately 90% of all participants were self-identified US citizens or permanent residents. Only one participant self-identified with an undocumented immigration status and this participant was in the Arizona-border sample (Table 1). The remaining 8% of participants had a temporary residency status, meaning that they were in the process of permanent residency status or had a border-crossing card, which allowed them to cross into the US and work in US agriculture. Transnational farmworkers were significantly more likely to be male, older and employed for more years in US agriculture compared to Arizona-based border farmworkers.

**Table 1:** **Demographic characteristics of Arizona-border and transnational farmworkers**.

	Total	Arizona border	Transnational	*p*-Value
	
	% (*n*/489)	% (*n*/349)	% (*n*/140)
Gender				
Female	40 (194/489)	47 (166/349)	20 (28/140)	**0.000**
Male	60 (295/489)	52 (183/349)	80 (112/140)	
Age, mean year (SD)	46 (11.2)	45 (10.8)	49 (11.0)	**0.001**
Immigration status				
US born or naturalized citizen	14 (66/484)	15 (52/344)	10 (14/140)	0.437
Permanent resident	80 (389/484)	79 (272/344)	84 (117/140)	
Temporary	6 (28/484)	6 (19/344)	6 (9/140)	
Undocumented	0.2 (1/484)	0.3 (1/344)	0 (0/140)	
Years in US agriculture, mean (SD)	19 (12.2)	19 (12.2)	21 (12.1)	**0.006**
Current health care coverage	57 (276/486)	55 (192/347)	60 (84/139)	0.313
Lacked coverage in last year	45 (151/332)	41 (92/225)	55 (59/107)	**0.018**

*Boldface p values indicate p < 0.05 from Fisher exact tests. Ns differ according to available data*.

### Experiences with US immigration officials, including local police

Although the Arizona-border sample was significantly more likely to see immigration officials in their neighborhoods, both study samples were as likely to observe immigration officials at the worksite, corner store, and the local supermarket. Arizona border respondents were significantly more likely to believe immigration officials, including local police, used individual characteristics of clothing and the type of vehicle to identify undocumented persons (Table 2). Although not statistically significant, Arizona border residents were more likely to be detained and questioned by local police regarding their immigration status compared to the transnational participants. Among those participants who were detained by local police, local police called immigration officials and detained Arizona and transnational farmworkers at almost equal rates.

**Table 2:** **Comparisons of experiences and encounters with US immigration and local police among Arizona-border and transnational farmworkers**.

	Total % (*n*/*N*)	Arizona border % (*n*/*N*)	Transnational % (*n*/*N*)	*p*-Value
Daily immigration official sightings in community settings (non-US port entry)	84 (373/443)	86 (285/330)	24 (88/113)	0.037
Neighborhood	68 (334/489)	89 (312/349)	16 (22/140)	**0.000**
Worksite	59 (287/489)	58 (203/349)	60 (84/140)	0.761
Corner store	20 (98/489)	20 (71/349)	19 (27/140)	0.901
Supermarket	42 (204/489)	44 (152/349)	37 (52/140)	0.224
Public bus	14 (70/489)	15 (53/349)	12 (17/140)	0.475
Characteristics used by immigration officials to identify undocumented persons				
Clothing	78 (382/487)	82 (284/348)	71 (98/139)	**0.010**
Type of car	70 (338/486)	73 (252/347)	61 (86/139)	**0.022**
Mexican appearance	65 (317/485)	67 (234/347)	60 (83/138)	0.139
Foreign-looking	65 (318/486)	68 (235/347)	60 (83/139)	0.113
Skin color	64 (311/486)	64 (223/347)	63 (88/139)	0.835
**Immigration detention experiences**				
Local police questioned immigration status, last 24 months	9 (43/489)	10 (36/349)	5 (7/140)	0.076
Local police called immigration	6 (20/346)	6 (16/269)	5 (4/77)	1.0
Detained by immigration	3 (12/348)	4 (10/272)	3 (2/76)	1.0
**Border community immigration stress scale (BCISS)^a^**				
Military patrolling the border	32 (154/484)	31 (108/348)	34 (46/136)	0.588
Encounters with local police	23 (113/487)	23 (81/348)	23 (32/487)	1.00
Encounters with immigration officials	20 (99/484)	19 (66/347)	24 (33/137)	0.214
**Reporting immigration encounters**				
Should report negative encounter	97 (471/482)	99 (341/346)	96 (130/136)	0.082
Knows how to report	33 (161/487)	34 (117/347)	31 (44/140)	0.667

*Boldface p values indicate p < 0.05 from Fisher exact tests*.

*^a^Frequency of intensely reported stressors from the border community and immigration stress scale (BCISS)*.

Almost all Arizona and transnational farmworkers believed negative immigration encounters should be reported. However, only about one-third of both populations reported knowing how to file a formal complaint of immigration mistreatment.

In terms of self-reported immigration-related intense stress, approximately one-third of all participants experienced intense stress due to military patrolling the border region. No <20% of all respondents experienced this same level of intense stress in anticipation of encounters with local police or encounters with immigration officials. There were no significant differences in the levels of stress produced by such encounters among the two samples.

### Complaint making regarding immigration mistreatment

Farmworker short narratives illuminated several themes regarding reasons to file a complaint of mistreatment by immigration officials (Table 3). Prevention of future mistreatment accounted for 29% of all narratives. According to farmworkers, filing a complaint of immigration-related mistreatment contributed to the prevention of mistreatment in several forms. First and foremost by filing a formal complaint one could contribute to raising awareness of immigration-related mistreatment. Complaints also served to elicit corrective action among those immigration officials who engaged in behavior beyond the scope of their mandate. More broadly, farmworkers believed formal complaints could contribute to the elimination of existing systems of discrimination.

**Table 3:** **Summary of reasons to file a formal complaint of immigration-related mistreatment among Arizona and transnational farmworkers of Mexican descent**.

	Illustrative quotes	
	Arizona farmworkers	Transnational farmworkers
**Prevention of future mistreatment**		
Prevent the abuse of others	Para evitar que vuelva a pasar una injusticia/to prevent an injustice from happening again	Para que ya no sigan abusando ni maltratando las personas/so they stop mistreating the people
Receive better treatment	Para evitar las injusticias para que no les pase lo mismo/to prevent injustice from happening so it does not happen Para prevenir maltratos en el future/to prevent future abuse Para que no nos sigan tratando mal a las personas/so they (immigration officals) will stop mistreating people Para tener un mejor trato!/to be treated better! Para ayudar a parar la discriminacion/To help stop discrimination Para que nos trate mejor y seamos escuchados/so we are treated better and they listen to us	
Raise awareness of immigration-related mistreatment	Para dar a saber las cosas que estan pasando/to make people aware of things that are occurring De esa forma dariamos a saber el maltrato que se les da a las personas/through this (formal complaint) we make known the mistreatment that they (immigration officials) enact on the people Para que se enteren los superiores de lo que esta pasando/so that the leadership or supervisors (immigration officals) know what is occurring	Para evitar las malos frutos/to eliminate the bad apples (immigration officals) Para que las autoridades mas altas se den cuenta de las injusticias que cometen/as so the upper level immigration officals understand the injustices that are being committed
Encourage corrective action	Para que un oficial abusivo sea castigado/so an abusive officer will be punished Para correjir el maltrato de los oficiales/to correct the abusive behavior of the officers No deben permitir que se maltrate a las personas/maltreatment should not permitted Para evitar las malos frutos/to remove the bad apples (immigration officials)	
Eliminate systems of discrimination	Para componer el sistema/to fix the system Para que no nos descriminen/so they (immigration officals) will stop discriminating us Para que haiga mas democracia en este lugar/so there is more democracy in this place’ Para que se acabe toda la discriminacion/to stop the discrimination Porque estan legales/because they [people being mistreated] are “legal” (authorized to be in the US)	Por injusticias por descriminacion/because of injustice and because of discrimination Si no comete uno algo malo, no tienen por que tratarnos mal/if you have not done anything wrong then they (immigration officials) have no reason to treat you badly Todas somas iguales; debemos ser tratados por igual/we are all equal and should be treated equally
**Protection and well-being**		
Recognition of rights	Porque tenemos derechos si tengamos documentos o no/because we have rights, whether we have papers (US citizenship or legal permanent residency documents) or not Porque somos personas igual que ellos/because we are people just like them (immigration officials) Porque roban a la gente de sus derechos/because they (immigration officials) rob the people of their rights Tenemos los mismos derechos que un ciudadano/because we have the same rights as a US citizen Todos somos legales somos humanos con los mismos derechos/because we are all authorized to be in the US with same rights (as any US citizen) Porque nadie tiene derecho de maltratarte/because no one has the right to mistreat you	Tenemos derechos y hay que reclamarlos/we have rights and we must reclaim these rights Por el humanismo; por que no debe haber injusticias/because of humanism, because there should not be such injustices Somos person/as y tambien tenemos derecho/we are people and we also have rights
Well-being of the individual and the collective community	Por el propio bien de uno/for ones own good	Por el bien de nosotros mismos/for the good of all of us
Individual and community resistance	Para que nos escuchen y no se nos siga ignorando/so they (immigration authority) listen to us and stop ignoring us) Para que nos respecten mas; y debemos defender nuestros derechos/so they (immigration authorities) respect us and we should defend out rights Deberiamos hablar para defender nuestros derechos; no ser atropellados/we should speak up to defend our rights, and not be overtaken	Para que sepan que la gente sabe sus derechos/so they (immigration authority) know that the people know their rights Por que debemos quejarnos, todos somos iguales/because we should complain, because we are all equal
**Mistreatment and abuse**		
	Es algo illegal si se debe reporter/its illegal (mistreatment) and it should be reported	Abuso de autoridad/abuse of power
	Porque en ocaciones abusan de las personas y los intimidan/because on occasions immigration officials abuse the people and intimidate them	Por violencia, abusos verbales,falta de respeto, abuso fisico/because of violence, verbal abuse, lack of respect, and physical abuse

The second major thematic category within the reasons to file a complaint of mistreatment was protection of overall well-being. Protection of well-being came in many forms including acknowledgment of civil and human rights, and avoidance of abuse. Farmworkers described in detail their inherent civil and human rights, which they believe should protect them and their community members from such mistreatment. Although far less mentioned, in some cases, farmworkers described the forms of resistance individual and community members engage in to monitor mistreatment.

When comparing the two groups, Arizona-border residents more often identified prevention of future mistreatment and human and civil rights compared to transnational participants who were more literal in their rational for complaint making who most often abuse of any kind. Both sets of participants reported formal complaint making about immigration-related mistreatment could contribute to positive changes in the larger immigration and police system.

We shift now to the reasons farmworkers would choose not to make a formal complaint of immigration-related mistreatment. Approximately 31% of the total sample stated fear as the number one reason not to file a formal complaint of mistreatment (Table 4). Fear came in many forms including fear of retaliation by immigration officials, fear of losing current immigration status, and fear of being deported (Table 4). Although a nuanced form of fear, other farmworkers described not filing a report because they wanted to avoid problems with officials, suggesting that by virtue of filing they may experience some form of investigation. Others expressed the sense that their complaint would not be taken seriously even if they reported it. The intensive work hours among farmworkers was also a deterrent from filing a report, as some farmworkers described not having enough time in the day to do so. This sense of not having enough time to file was often linked to the idea of wasting time in filing as if their complaint would not be acted upon.

**Table 4:** **Summary of reasons not to file a formal complaint of immigration-related mistreatment among Arizona and transnational farmworkers of Mexican descent**.

	Illustrative quotes	
	
	Arizona Farmworkers	Transnational Farmworkers
**General fear**		
Fear of retaliation	Por miedo a que tomen la queja en nuestra contra/for fear they (immigration officials) will use the complaint against us	Por miedo a que haiga represarios/for fear of retaliation
	Por miedo a en contrarse nuevamente con la persona que lo maltrato/for fear of encountering the person (immigration officer) who mistreated you	Por miedo a tener otro problema mas uno nunca sabe si al hacer una queja como te vaya/for fear of having one more problem because you do not know how making a complaint with effect you later
	Porque las personas tienen miedo a lo que pueda pasar despues no conocen las leyes/because the people are afraid of what could happen after (they make a complaint) because they do not know the law	Por miedo despes vayan a decir el nombre de quien los dijo relaliaton against person on his family/out of fear they (immigration officials) may say the name of the person who complained and they will retaliate against your family
	Por precaucion a lo que pueden hacer en contra de la familia (represalias)/out of precaution because of what could potentially happen to the family (retaliation)
Fear of losing current immigration status	Por miedo a que les quiten los papeles/for fear they (immigration) will take away legal documents	Por miedo a perder su estatus migratorio/out of fear that you might lose your immigration status
	Por miedo a perder papeles o a ser ignorados/for fear of losing papers (legal immigration papers) or be ignored	
Fear of being deported	Por miedo a que los deporten/for fear of being deported	Por miedo a una deportacion/fear of being deported
	Por miedo, a que los detengan o los deporte/for fear that you will be detained and deported
	Por miedo de que los manden para Mexico o que no los tomen en cuanta/for fear they will send you to Mexico or they will not take your complaint seriously
**Other themes**		
Desire to avoid problems	Por miedo a enfrentarse a si mismo rasismo/for fear of confronting the same type of racism	Por miedo o simplemente no quiere uno meterse en problemas/for fear of simply not wanting to become involved in problems
Waist of time	El miedo a perder tiempo y papeles y dinero por dejar de trabajar/the fear of losing time, your papers, and money because you had to leave work	Por que nunca hacen nada las autoridades/because the authorities will never do anything
	Piensan que no se les va a hacer caso… como que no vale la pena/the people think that the immigration officals are not going to do anything and it is not worth making a complaint	Por miedo a que no hagan caso o no te tomen en cuenta/the fear of no one doing anything and not taking your complaint seriously
Not enough time	Por no perder el tiempo de trabajar y las vueltas que tendrian que dares/to not loose time at work with all the paperwork you will have to do	
	Por falta de tiempo; sale uno bien cansado y pensado que va a ser ignorado si va/Due to lack of time, you leave work so tired, and think you will be ignored if you go (to make a complaint)	
Rights	Por miedo por pensar que no tiene el derecho de reclamar/the fear that one thinks they do not have the right to complain Porque las personas no se sienten con el valor de hacerlo/because people may not have the courage to make a complaint	Porque si uno no ha hecho nada incorrecto y tiene sus documentos en regla las autoridades no deben de maltratara las personas/because if one has not done anything wrong and you have your papers in order the authorities should not be mistreating people

## Discussion

We show that in the border region, immigrants and migrants of Mexican descent with US permanent residence and citizenship feel vulnerable to being identified as “out of place” and, subsequently, the target of immigration enforcement. Immigration officials’ presence was pervasive and not confined to the US port of entry but was experienced by participants in public spaces, including neighborhoods, worksites, and local markets. Arizona border and transnational immigrant and migrant farmworkers experience high levels of stress associated with encounters and or anticipated encounters with immigration officials. Furthermore, participants believed that these officials used personal characteristics to differentiate the population and identify individuals with an undocumented or “illegal” immigration status. We were unable to confirm our hypothesis, as there were only a few consistent differences between the two samples that would suggest that any one group would internalize “illegality” more or less than the other. Lack of difference between the two groups suggests that US immigration enforcement permeates the public spaces where both Arizona resident and transnational farmworkers conduct their lives constituting an imminent threat of state-sponsored violence to both of these authorized populations.

Most notable of the ways in which the two populations may internalize a sense of “illegality” or “undeservingness” for social protection from immigration-related discrimination and mistreatment is the high proportion of respondents reporting fear as the primary reason why they themselves or others in the community may not report immigration mistreatment. Immigration enforcement in the borderlands relies heavily not only on undocumented status but also on legal status as perceived through ethno-racial profiling of subjects. In the context of militarized border enforcement and the criminalization of immigration, the distinctions between rights-bearing subjects and those without any rights are blurred. While farmworkers indicate that they know their rights to file complaints and the positive potential of doing so (Table 3), their fears indicate that they do not believe their rights can protect them within the militarized climate of the border (Table 4). Permanent residents and citizens of Mexican descent internalize their subordinated racialized status, fearing that their legal status can be easily revoked if they file complaints of maltreatment by immigration officers or local police. Deportability – an essential dimension of “illegality” – is not only implicated in creating an exploitable workforce (5) but also is a key site of the production of state power and the ability of the US to govern its citizens and permanent residents (37). The social cost of the symbolic and material fortification of the border can be measured in its effects upon farmworkers’ sense of exclusion and fear of losing “that which has been established,” that is, their basic rights as residents and citizens. This study provides further evidence of the “spillover” effects of immigration enforcement onto groups who are not the target of immigration enforcement. The resulting biopsychological harm demonstrates how the current enforcement regime is detrimental to society (38).

### Public health policy implications and future research

As border security remains compulsory to the US immigration reform policy debate, and persuasive in public discourse, our study confirms that immigration policy and specifically those polices aimed at border enforcement is a structural determinant of health. Defined by the WHO Commission on Social Determinants of Health, structural determinants are those distal policy and systems levels phenomena that directly and indirectly affect the public’s health (39, 40). Such structural determinants require large-scale political and social change. Institutional practices of discrimination within US immigration and border enforcement political systems have only recently emerged as determinants of health inequality (41) and few studies have linked these experiences to poor mental health outcomes (33). Broadly, restrictive or punitive immigration policies are known to limit access to health and social services (42, 43), education opportunities, and adequate employment remuneration (41, 44). In Arizona, anti-immigrant policies have been documented to limit mobility among Mexican immigrants to engage in normal activities and create fear of accessing health and social services among the population (43).

Our study provides strong evidence for the Department of Homeland Security (DHS) to enact and enforce policies that benefit public health, such as; (1) articulate and make transparent CBP training, oversight, investigation protocols, and the disciplinary actions taken against CBP officers and local police who breach their scope when enforcing immigration law (20, 45); (2) create a transparent, community-centered oversight system to document and monitor immigration-related victimization, including corruption and excessive use of force by CBP and local police enacting immigration law; (3) develop an accountability plan by CBP and local law enforcement to systematically report and respond to community concerns of corruption and excessive use of force.

Participatory action research that fully engages affected border communities is necessary to monitor immigration-related victimization and locate the points of community and policy-level intervention to decrease victimization within border communities. The Southern Border Communities Coalition’s, “Revitalize, Not Militarize” is one example of a grassroots effort in which border community members have mobilized to reframe the issue of border security as an issue of economic development. Calling for investment in all border communities to improve the quality of life of the region and trade between the US and Mexico, the campaign engages a multi media platform for border residents to share their testimonies, monitor immigration-related mistreatment, and civil and human rights abuses and advocate at state and national levels for oversight and accountability by the Department of Homeland Security and Customs and Border Patrol Agents (46). Such community-driven campaigns linked to advocacy can contribute to empowerment of affected communities and have the potential to begin to repair the detrimental effects of immigration-related structural vulnerability, which includes the internalization and normalization of such violence.

### Limitations and strengths

This study may not be generalizable to non-border communities; study participants may be more likely to be in frequent contact with immigration authorities compared to those individuals in non-border communities. These results may also underestimate the prevalence of immigration-related mistreatment and associated stress in highly militarized and policed communities, as those individuals with an undocumented immigration status may be less likely to participate. Data are self-report and the potential for social desirability may also contribute to over or under estimation of mistreatment experiences. The CBPR approach, however, contributed to the overall strength of the study, specifically, in survey development, data collection, and the validity of the study constructs to community identified health issues. Study partners were uniquely embedded in the community, and shared many of the cultural and immigration trajectories of study participants thus giving UA researchers invaluable insight and access to a highly vulnerable population. This historical and trusting relationship between University researchers and study partner agencies, and the utilization of *Promotoras* as primary data collectors contributed to increased cultural salience of sampling procedures, survey instrument development and implementation as evidenced by a 93% response rate and limited missing data in the household survey data.

## Conclusion

US citizens and permanent residents of Mexican descent living in the border region experience frequent encounters with immigration officials in public spaces at almost equal rates. These encounters are not confined to the point of entry. Anticipation of such encounters is experienced as intense stressors. Moreover, the primary reason for not reporting immigration-related abuse or mistreatment is fear and specifically the fear of losing existing immigration status. Such mistrust in the system and fearing retaliation by the state is evidence of a population who has potentially normalized mistreatment as a form of coping in the face of a broken system in which justice and retribution could only occur at a cost. Multi-institutional and community-centered systems for reporting and mitigating immigration-related victimization are required. Applied participatory research with affected communities can mitigate the public health effects of state-sponsored immigration discrimination and violence.

## Conflict of Interest Statement

The authors declare that the research was conducted in the absence of any commercial or financial relationships that could be construed as a potential conflict of interest.

## References

[1] Meissner D Kerwin D Chishti M Bergeron C Immigration Enforcement in the United States: The Rise of a Formidable Machinery. Washington, DC: Migration Policy Institute (2013).

[2] Dunn T The Militarization of the U.S.-Mexico Border, 1978-1992: Low-Intensity Conflict Doctrine Comes Home. Austin, TX: CMAS Books, University of Texas at Austin (1996).

[3] Heyman J The state and mobile peoples at the U.S.-Mexico border. In: Lem W Gardiner Barber P, editors. Class, Contention, and a World in Motion. Oxford: Berghahn Press (2010). p. 58–78.

[4] Sabo S Shaw S Ingram M Teufel-Shone N Carvajal S de Zapien JG Everyday violence, structural racism and mistreatment at the US-Mexico border. Soc Sci Med (2014) 109:66–74. 10.1016/j.socscimed.2014.02.005 24705336

[5] De Genova N Working the Boundaries: Race, Space, and “Illegality” in Mexican Chicago. Durham, NC: Duke University Press (2005).

[6] Menjívar C Liminal legality: Salvadoran and Guatemalan immigrants’ lives in the United States. Am J Sociol (2006) 111(4):999–1037. 10.1086/499509

[7] Chavez L The Latino Threat: Constructing Immigrants, Citizens, and the Nation. Stanford, CA: Stanford University Press (2008).

[8] Ngai M Impossible Subjects: Illegal Aliens and the Making of Modern America. Princeton, NJ: Princeton University Press (2004).

[9] Bourgois P Recognizing invisible violence: a thirty-year ethnographic retrospective. In: Rylko-Bauer B Whiteford L Farmer P, editors. Global Health in Times of Violence. Santa Fe, NM: School for Advanced Research Press (2009). p. 17–40.

[10] Bourdieu P Wacquant L Symbolic violence. In: Scheper-Hughes N Bourgois P, editors. Violence in War and Peace: An Anthology. Malden, MA: Blackwell (2004). p. 272–4.

[11] Coutin SB Legalizing Moves: Salvadoran Immigrants Struggle for U.S. Residency. Ann Arbor, MI: University of Michigan Press (2000).

[12] Coutin SB Being en route. Am Anthropol (2005) 107(2):195–206. 10.1525/aa.2005.107.2.195

[13] Coutin SB Denationalization, inclusion, and exclusion: negotiating the boundaries of belonging. Indiana J Global Leg Stud (2000) 7(2):585–93.

[14] Golash-Boza TM Immigration Nation: Raids, Detentions and Deportations in Post 9/11 America. Boulder, CO: Paradigm Publishers (2012).

[15] Martinez D Slack J Heyman JM Bordering on Criminal: The Routine Abuse of Migrants in the Removal System. Part I: Migrant Mistreatment While in U.S. Custody. Washington, DC: American Immigration Council (2013).

[16] Nevins J Operation Gatekeeper and Beyond: The War on “Illegals” and the Remaking of the U.S.-Mexico Boundary. 2nd ed New York, NY: Routledge (2010).

[17] Coleman M Immigration geopolitics beyond the Mexico-US border. Antipode (2007) 39(1):54–76. 10.1111/j.1467-8330.2007.00506.x

[18] Golash-Boza TM Due Process Denied: Detentions and Deportations in the United States. New York, NY: Routledge (2012).

[19] Stumpf J The crimmigration crisis: immigrants, crime, and sovereign power. Am Univ Law Rev (2006) 56(2):367–419.

[20] Slack J Martinez D Whiteford S Lee A Border Militarization and Health: Violence, Death, and Security (2013). Available from: http://works.bepress.com/scott_whiteford/ (accessed 9, 2015)

[21] Viruell-Fuentes EA. Beyond acculturation: immigration, discrimination, and health research among Mexicans in the United States. Soc Sci Med (2007) 65:1524–35. 10.1016/j.socscimed.2007.05.010 17602812

[22] Quesada J Kain Hart L Bourgois P. Structural vulnerability and health: Latino migrant laborers in the United States. Med Anthropol (2011) 30(4):339–62. 10.1080/01459740.2011.576725 21777121 PMC3146033

[23] Gonzales RG Chavez L “Awakening to a nightmare”: abjectivity and illegality in the lives of undocumented 1.5-generation Latino immigrants in the United States. Curr Anthropol (2012) 53(3):255–81. 10.1086/665414

[24] Lee A “Illegality,” health problems and return migration: cases from a migrant sending community, Puebla, Mexico. Reg Cohes (2013) 3(1):62–93. 10.3167/reco.2013.030104

[25] McGuire S Georges J. Undocumentedness and liminality as health variables. ANS Adv Nurs Sci (2003) 26(3):185–95. 10.1097/00012272-200307000-00004 12945654

[26] Willen SS Toward a critical phenomenology of “illegality”: state power, criminalization, and abjectivity among undocumented migrant workers in Tel Aviv, Israel. Int Migr (2007) 45(3):8–38. 10.1111/j.1468-2435.2007.00409.x

[27] Holmes S Fresh Fruit, Broken Bodies. Oakland, CA: University of California Press (2014).

[28] Villarejo D Lighthall D Williams D Souter A Mines R Suffering in Silence: A Report on the Health of Agricultural Workers. Berkeley, CA: California Institute for Rural Studies (2000).

[29] Villarejo D McCurdy S Bade B Samuels S Lighthall D Williams D. The health of California immigrant hired farmworkers. Am J Ind Med (2010) 53:387. 10.1002/ajim.20796 20191600

[30] Alderete E Vega WA Kolody B Aguilar-Gaxiola S. Lifetime prevalence of and risk factors among Mexican migrant farmworkers in California. Am J Public Health (2000) 90:608. 10.2105/AJPH.90.4.608 10754977 PMC1446194

[31] Hovey J Mental Health and Substance Abuse Migrant Health Monograph Series. 4th ed Buda, TX: National Center for Farmworker Health Inc (2001). p. 18–35.

[32] Rosales C Carvajal S McClelland J Ingram M Rubio-Goldsmith R de Zapien J Challenges to Farmworker Health at the US Mexico Border: A Report on Health Status of Yuma County Agricultural Workers. Tucson, AZ: University of Arizona (2009).

[33] Carvajal SC Rosales C Rubio-Goldsmith R Sabo S Ingram M McClelland DJ The border community and immigration stress scale: a preliminary examination of a community responsive measure in two southwest samples. J Immigr Minor Health (2012) 15(2):427–36. 10.1007/s10903-012-9600-z 22430894 PMC4431619

[34] Koulish RE US Immigration Authorities and Victims of Human and Civil Rights Abuses: The Border Interaction Project Study of South Tucson, Arizona, and South Texas. Tucson, AZ: Mexican American Studies & Research Center, University of Arizona (1994).

[35] Goldsmith PR Romero M Rubio-Goldsmith R Escobedo M Khoury L Ethno-racial profiling and state violence in a Southwest barrio. Aztlán (2009) 34(1):93–123.

[36] Patton M Qualitative Research and Evaluation Methods. 3rd ed Thousand Oaks, CA: Sage (2002).

[37] De Genova N The deportation regime: sovereignty, space, and the freedom of movement. In: De Genova N Peutz N, editors. The Deportation Regime. Durham, NC: Duke University Press (2010). p. 33–65.

[38] Aranda E Menjívar C Donato K The spillover consequences of an enforcement-first U.S. immigration regime. Am Behav Sci (2014) 58(13):1687–95. 10.1177/0002764214537264

[39] Marmot M Allen J Bell R Goldblatt P. Building of the global movement for health equity: from Santiago to Rio and beyond. Lancet (2011) 379(9811):181–8. 10.1016/S0140-6736(11)61506-7 22014678

[40] CSDH. Closing the Gap in a Generation: Health Equity Through Action on the Social Determinants of Health. Final Report of the Commission on Social Determinants of Health. Geneva (2008). 10.1016/S0140-6736(08)61690-6 18994664

[41] Acevedo-Garcia D Sanchez-Vaznaugh EV Viruell-Fuentes EA Almeida J. Integrating social epidemiology into immigrant health research: a cross-national framework. Soc Sci Med (2012) 75(12):2060–8. 10.1016/j.socscimed.2012.04.040 22721965

[42] Hacker K Chu J Leung C Marra R Pirie A Brahimi M The impacts of immigration and customs enforcement on immigration health: perceptions of immigrants in Everette, Massachusetts, USA. Soc Sci Med (2011) 73:586–94. 10.1016/j.socscimed.2011.06.007 21778008 PMC3159749

[43] Hardy L Getrich C Quezada J Guay A Michalowski R Henley E. A call for further research on the impact of state level immigration policies on public health. Am J Public Health (2012) 102(7):1250–4. 10.2105/AJPH.2011.300541 22594736 PMC3477996

[44] Viruell-Fuentes EA “Its so hard”: racialization processes, ethnic identity formations, and their health implications. Du Boise Rev Washington, DC: American Immigration Council (2011) 8(1):37–52. 10.1017/S1742058X11000117

[45] Martínez D Slack J Heyman J “Migrant Mistreatment While in U.S. Custody.” Part I of Bordering on Criminal: The Routine Abuse of Migrants in the Removal System Washington, DC: American Immigration Council (2013).

[46] Sustainable Business Coalition. Revitalize, not Militarize Campaign (2014). Available from: http://www.revitalizenotmilitarize.org/

